# Prednisolone Suppresses the Extracellular Release of HMGB-1 and Associated Inflammatory Pathways in Kawasaki Disease

**DOI:** 10.3389/fimmu.2021.640315

**Published:** 2021-05-17

**Authors:** Kentaro Ueno, Yuichi Nomura, Yasuko Morita, Yoshifumi Kawano

**Affiliations:** ^1^ Department of Pediatrics, Kagoshima University Graduate School of Medical and Dental Sciences, Kagoshima, Japan; ^2^ Department of Pediatrics, Kagoshima City Hospital, Kagoshima, Japan

**Keywords:** pediatrics, Kawasaki disease (KD), DAMPs (damage-associated molecular patterns), prednisolone, high mobility group box-1

## Abstract

Innate immune activity plays an essential role in the development of Kawasaki disease (KD) vasculitis. Extracellular release of high mobility group box-1 (HMGB-1), an endogenous damage-associated molecular pattern protein that can activate the innate immune system and drive host inflammatory responses, may contribute to the development of coronary artery abnormalities in KD. Prednisolone (PSL) added to intravenous immunoglobulin treatment for acute KD may reduce such abnormalities. Here, we evaluate the dynamics of HMGB-1 and therapeutic effects of PSL on HMGB-1-mediated inflammatory pathways on KD vasculitis *in vitro*. Serum samples were collected prior to initial treatment from patients with KD, systemic juvenile idiopathic arthritis (sJIA), and from healthy controls (VH), then incubated with human coronary artery endothelial cells (HCAECs). Following treatment of KD serum-activated HCAECs with PSL or PBS as a control, effects on the HMGB-1 signaling pathway were evaluated. Compared to that from VH and sJIA, KD serum activation induced HCAEC cytotoxicity and triggered extracellular release of HMGB-1. KD serum-activated HCAECs up-regulated extracellular signal-regulated kinase (ERK)1/2, c-Jun N-terminal kinase (JNK) and, p38 phosphorylation in the cytoplasm and nuclear factor kappa B (NF-κB) phosphorylation in the nucleus and increased interleukin (IL)-1β and tumor necrosis factor (TNF)-α production. PSL treatment of KD serum-activated HCAECs inhibited extracellular release of HMGB-1, down-regulated ERK1/2, JNK, p38, and NF-κB signaling pathways, and decreased IL-1β and TNF-α production. Our findings suggest that extracellular HMGB-1 plays an important role in mediating KD pathogenesis and that PSL treatment during the acute phase of KD may ameliorate HMGB-1-mediated inflammatory responses in KD vasculitis.

## Introduction

Kawasaki disease (KD), an acute systemic vasculitis of unknown etiology, commonly occurs in children (1), and can ultimately lead to complex coronary artery abnormalities (CAAs). Despite standard treatment with high-dose intravenous immunoglobulin (IVIG) and aspirin, approximately 10% to 20% of patients experience persistent or recurrent fever and appear to have elevated risk of developing CAAs (2, 3). Notably, combined treatment with prednisolone (PSL) is more effective in preventing CAAs than IVIG alone in non-responders, thereby reducing the need for additional rescue treatments (4). Thus, corticosteroid combination therapy is considered a promising pre-emptive primary treatment (5, 6).

Innate immune activity is integral toward KD vasculitis etiology (7). High mobility group box-1 (HMGB-1), a representative damage-associated molecular pattern (DAMP) protein, plays a central role in regulating programmed cell death and survival (8). As sentinel innate immune cells, endothelial cells release DAMPs as endogenous danger signals that alert the innate immune system to unscheduled cell death, microbial invasion, and stress (9). Extracellular HMGB-1 coordinates cellular responses associated with immune system activation, cell migration, cell growth, and tissue repair and regeneration, and binds to receptors, such as receptor for advanced glycation end products (RAGE) and Toll-like receptors (TLRs) to activate proinflammatory responses. Downstream signaling involving mitogen-activated protein kinase (MAPK) such as extracellular signal-related kinase (ERK)/c-Jun N-terminal kinase (JNK)/p38 and nuclear factor kappaB (NF-κB) facilitates cellular responses including inflammatory cytokine, chemokine, and corresponding receptor expression (10). Injury-evoked increased HMGB-1-mediated inflammatory responses can increase cardiovascular disease severity (11–14). Notably, serum HMGB-1 and S100 protein levels are also elevated in patients in the acute phase of KD (15–17). Moreover, Nucleotide-binding oligomerization domain-like receptor family, pyrin domain-containing 3 (NLRP3)-dependent endothelial cell pyroptosis *via* HMGB-1/RAGE/cathepsin B signaling may contribute to coronary artery endothelial cell (CAEC) damage in KD vasculitis (18). Thus, DAMP-mediated innate immune system activation may facilitate pathological inflammatory responses in KD vasculitis.

We hypothesize that PSL treatment, a standard anti-inflammatory therapy, may suppress inflammation in KD by reducing inflammatory cytokines and DAMPs produced by CAECs consequent to acute KD vasculitis. In this study, we clarified the role of HMGB-1 and evaluated the *in vitro* therapeutic effects of PSL on HMGB-1-mediated inflammatory responses in CAECs during acute KD vasculitis.

## Materials and Methods

### Patients

The study was approved by the Kagoshima University and Kagoshima City Hospital Ethics Committee and performed in accordance with the International Conference on Harmonization guidelines for Good Clinical Practice and the Declaration of Helsinki (approval number: MD26-156, Approval date: January 13, 2016). We enrolled eight consecutive patients undergoing treatment for KD at the host institution applying the following exclusion criteria: 1) cardiovascular disease, hematological disease, congenital malformations, primary disease of major organs, and genetic/chromosomal abnormalities; 2) bacteremia or sepsis with positive blood culture; 3) recurrent KD symptoms; 4) previous use of corticosteroids or immunosuppressive treatment; 5) development of coronary artery lesions. We used four healthy subjects and four patients with systemic juvenile idiopathic arthritis (sJIA) as vehicle and disease controls, respectively. Written informed consent was obtained from the parents of all study participants and serum samples were collected from the patients and healthy subjects.

KD was defined using the Japanese criteria (19). The first day of illness was defined as the first day of fever. Treatment was initiated when KD was considered highly likely even if all KD criteria were not met. Patients with KD received a single IVIG infusion (2 g/kg) together with aspirin (30 mg/kg/day, decreased to 3 to 5 mg/kg/day if afebrile for ≥ 28 days following fever onset).

### Blood-Sample Collection

Serum samples were collected from patients with KD and sJIA before initial treatment and from healthy controls, separated by centrifugation (700 × *g*, 15 min), and stored at −40°C.

### Endothelial Cell Culture and Preparation

Primary human CAECs (HCAECs) were purchased from PromoCell (Heidelberg, Germany) and cultured using MV 2 kit endothelial cell growth medium (PromoCell). Medium was changed every 24 h. Cells at 70% to 80% confluence were seeded into 96-, 8-, or 6-well microplates for assays and fluorescence microscopy. Third passage HCAECs were used for experiments. Vehicle (VH) and KD controls comprised HCAECs at 90% confluence incubated for 24 h in MV 2 basal medium (PromoCell) with 7.5% healthy volunteer or KD patient serum, respectively. Serum-activated HCAECs treated with phosphate buffered saline (PBS) and PSL (10^−6^ M/well (20); Shionogi, Osaka, Japan) for 24 h were defined as KD+PBS and KD+PSL HCAECs, respectively. After each experiment, media were replaced with serum-free fresh MV 2 growth media to discriminate serum cytokine effects. ELISA evaluation of final washes demonstrated TNF-α levels (R&D systems, Minneapolis, MN, USA) below detectable limits (< 5.5 pg/mL) (**Supplementary Figure 1**). Experiments were repeated at least twice and media were maintained between pH 7.2 and 7.4.

### Analysis of Serum-Activated HCAEC Viability and Cytotoxicity

Cell viability was measured using 3-(4,5-dimethylthiazol-2-yl)-2,5-diphenyl tetrazolium bromide (MTT) assay (Dojindo, Kumamoto, Japan). HCAECs were cultured in growth medium in 96-well plates (0.5 × 10^4^ cells/well). Following each experiment, medium was replaced with fresh MV 2 basal medium and the final sample volume adjusted to 100 μL/well. Samples were subjected to MTT assay according to manufacturer instructions and measured in duplicate, using Microplate Reader (Tecan Infinite M200).

Cytotoxicity was evaluated *via* fluorescence to measure the activity of dead-cell protease, which is released from cells with impaired membrane integrity, using a CytoTox-Glo cytotoxicity assay (Promega, Madison, WI, USA) according to manufacturer instructions. Briefly, HCAECs (0.5 × 10^4^ cells/well) were cultured in growth medium in 96-well plates. After each experiment, medium was replaced with fresh MV 2 basal medium (100 μL/well final volume). Fluorescence measured using a Tristar multimode microplate reader (LB 941; Berthold Technologies, Oak Ridge, TN, USA), was directly proportional to the number of dead cells. Each sample was measured in duplicate.

### Assessment of Extracellular HMGB-1 Released From KD Serum-Activated HCAECs

HMGB-1 content in supernatant released from KD serum-activated HCAECs for 24 h was measured in duplicate using a commercial ELISA kit (Shino-Test Corporation, Tokyo, Japan) according to manufacturer instructions. The minimum HMGB-1 detection value was 1.0 ng/mL.

### Quantitative Analysis of Receptors on KD Serum-Activated HCAECs

Total RNA samples were extracted from cell lysates of serum-activated HCAECs using the RNeasy Mini Kit (#74104; QIAGEN, Hilden, Germany) according to manufacturer’s instructions. Reverse transcription was performed using a PrimeScript RT Reagent Kit (TaKaRa, Tokyo, Japan) according to manufacturer’s instructions. An equivalent volume of cDNA solution was used for real-time PCR quantification using a Thermal Cycler Dice Real Time System (TaKaRa), with glyceraldehyde-3-phosphate dehydrogenase (*GAPDH*) as the internal standard. At least two biological replicates were performed and specific PCR product amplification was confirmed by melting curve analysis. Gene expression was calculated using the 2^−ΔΔCT^ method. **Table 1** lists primer sequences (*GAPDH*, *RAGE*, *TLR2*, and *TLR4*) and RT-PCR conditions.

**Table 1:** Primer sequences and PCR conditions.

mRNA	Primer sequences	Annealing time and temperature (°C)	Cycle no.	Fragment length/base pairs
*GAPDH*	sense: 5′-GCACCGTCAAGGCTGAGAAC-3′	0.5 min; 95	40	138
	antisense: 5′-TGGTGAAGACGCCAGTGGA-3′			
*RAGE*	sense: 5′-GGAAAGGAGACCAAGTCCAA-3′	1 min; 59	30	166
	antisense: 5′-CATCCAAGTGCCAGCTAAGA-3′			
*TLR2*	sense: 5′-GGCTTCTCTGTCTTGTGACC-3′	0.5 min; 49	32	294
	antisense: 5′-GGGCTTGAACCAGGAAGACG-3′			
*TLR4*	sense: 5′-TTGTATTCAAGGTCTGGCTGG-3′	0.5 min; 47	32	438
	antisense: 5′-GCAAACCTTTGAAACTCAAGCC-3′			

GAPDH, Glyceraldehyde-3-phosphate dehydrogenase; RAGE, receptor for advanced glycation end products; TLR2, Toll-like receptor 2; TLR4, Toll-like receptor 4. Bold values indicate statistically significance in patient characteristics between KD group and sJIA group.

### Soluble RAGE (sRAGE) Production

Supernatant sRAGE levels released from KD serum-activated HCAECs for 24 h were measured in duplicate using a commercially available ELISA kit (R&D Systems) according to manufacturer instruction. The minimum sRAGE detection value was 4.21 pg/mL.

### Immunofluorescence Staining for HMGB-1

HCAECs prepared on 8-well imaging chamber (7.0 × 10^4^ cells/well) were incubated with MV 2 growth medium (37°C, 5% CO_2_). VH, KD control, KD+PBS, and KD+PSL HCAECs were washed with PBS, fixed, permeabilized using the Image-iT fixation/permeabilization kit (Invitrogen, Grand Island, NY, USA) and intracellularly stained with Alexa Fluor 594 anti-HMGB-1 (red) in blocking buffer overnight at 4°C. Nuclei were counterstained using ProLong Gold Antifade reagent with 4′,6-diamidino-2-phenylindole (DAPI, blue) (Life Technologies, Eugene, OR, USA). Randomly selected cells (*n* = 100) from each group were observed using fluorescence microscopy (Keyence BZ-X700; Carl Zeiss, Oberkochen, Germany). Quantitative analysis of immunofluorescence staining was performed using ImageJ software (National Institutes of Health, Bethesda, MD, USA) (21).

### Western Blot Analysis

Third-passage HCAECs were cultured in 6-well plates (4.0 × 10^5^ cells/well) with growth medium. After each experiment, adherent cells were lysed using the total protein extraction kit for animal cultured cells and tissues (Invent Biotechnologies, Plymouth, MN, USA) and prepared for immunoblotting. Each 15 μL sample was subjected to 10% gradient SDS-PAGE and electrotransferred onto a polyvinylidene difluoride membrane, then immunoblotted using primary antibodies against phosphorylated ERK 1/2 (p-ERK; Cell Signaling Technology (CST), Beverly, MA, USA; 1:2,000 dilution), ERK 1/2 (CST; 1:1,000), phosphorylated stress-activated protein kinase (SAPK)/c-Jun amino terminal kinase (JNK) (CST; 1:1,000), SAPK/JNK (CST; 1:1,000), phosphorylated p38 (CST; 1:1,000), p38 (CST; 1:1,000), anti-NLRP3 (CST; 1:1,000), anti-cleaved Caspase-1, (CST; 1:1,000), IL-1β (CST; 1:1,000), and TNF-α (CST; 1:1,000), followed by horseradish peroxidase-conjugated secondary antibody (Medical & Biological Laboratories, Nagoya, Japan; 1:1,000). For NF-κB p65, nuclear and cytoplasmic proteins were extracted using an extraction kit (SC-003, Invent Biotechnologies) according to manufacturer instructions. Each 15 μL sample was subjected to 10% gradient SDS-PAGE and electrotransferred onto a polyvinylidene difluoride membrane and immunoblotted using primary antibodies against phosphorylated NF-κB p65 (p-NF-κB p65; CST; 1:1,000) or NF-κB p65 (CST; 1:1,000), followed by horseradish peroxidase-conjugated secondary antibody (Medical & Biological Laboratories; 1:1,000). Housekeeping protein, such as β-actin and glyceraldehyde-3-phosphate dehydrogenase (GAPDH) were used as loading controls on the account of their expression levels. Proteins were visualized by chemiluminescence with SignalFire ECL Reagent (CST), and quantified using Fluor Chem FC2 (Alpha Innotech Kasendorf, Germany). All blotting experiments were repeated at least twice.

### Statistical Analysis

Continuous variables are reported as median values with interquartile ranges (IQR; 25th–75th percentiles). Categorical variables are presented as frequencies and percentages. Baseline comparisons between patients were performed using Student’s t-tests, Mann–Whitney *U*-tests, or χ^2^ analysis (with Yates’ correlation or Fisher’s exact test, as appropriate). Differences between > 2 groups were evaluated by one-way ANOVA followed by the Bonferroni or Games–Howell test and the Kruskal–Wallis test with the Dunn’s *post hoc* test. The former was performed when the variables showed a normal distribution; otherwise, the latter was used. All statistical analyses were performed using SPSS statistical software (v.25.0; SPSS Japan Inc., Tokyo, Japan). A two-tailed *P* < 0.05 was considered statistically significant.

## Results

### Clinical Characteristics and Laboratory Findings in Patients With KD and sJIA

Sera from eight patients with KD (median, 1.6 years) and four with sJIA (median 7.8 years) were evaluated. **Table 2** lists patient clinical characteristics and laboratory findings. Age and body weight significantly differed between the groups as KD and sJIA patients exhibited different peak ages of onset. Laboratory data, including baseline white blood cell counts, neutrophil counts, and C-reactive protein levels, did not significantly differ with the exception of total protein and sodium levels.

**Table 2:** Patient characteristics between Kawasaki disease (KD) and systemic juvenile idiopathic arthritis (sJIA).

Group	KD (n = 8)	sJIA (n = 4)	*P* value
Male, N (%)	5 (62.5)	2 (50.0)	0.692
Age at onset (years)	1.6 (0.5–2.9)	7.8 (4.6–13.3)	**0.011**
Body weight (kg)	10.8 (7.8–13.0)	17.7 (13.0–25.1)	0.061
White blood cell count (×10^3^/μL)	15.8 (11.9–18.1)	14.6 (12.1–24.4)	1.000
Neutrophil count (×10^3^/μL)	11.7 (7.9–14.5)	11.7 (10.0–20.0)	0.734
Platelet count (×10^4^/μL)	32.8 (28.3–41.5)	59.0 (34.6–66.4)	0.089
Aspartate aminotransferase (IU/L)	45 (26–288)	29 (23–33)	0.202
Alanine aminotransferase (IU/L)	56 (10–336)	14 (10–21)	0.348
Lactate dehydrogenase (IU/L)	336 (273–394)	353 (283–494)	0.610
Total protein (g/dL)	6.6 (6.4–7.0)	7.7 (7.3–7.8)	**0.006**
Albumin (g/dL)	3.4 (3.2–3.7)	3.3 (3.1–3.5)	0.330
Sodium (mEq/L)	134 (129–134)	138 (137–142)	**0.006**
C-reactive protein (mg/dL)	6.9 (4.4–10.5)	6.9 (5.3–9.0)	1.000

Data are expressed as median values and interquartile range (25th, 75th percentile), or number (proportion, %). Bold values indicate statistically significance in patient characteristics between KD group and sJIA group.

### Serum Concentration From Patients With KD Necessary to Serum-Activate HCAECs

To determine the serum concentration from patients with KD necessary to exert cytotoxic effects on HCAECs, we first exposed HCAECs to four independent KD sera concentrations, 0% (untreated), 5%, 7.5%, and 12.5%, for 24 h. KD serum induced significant cytotoxic effects on HCAECs at concentrations ≥ 7.5% (**Supplementary Figure 2A**) while maintaining cellular viability (**Supplementary Figure 2B**). Therefore, 7.5% KD serum was used for subsequent *in vitro* experiments.

### Proliferative Activity and Cytotoxicity of Serum-Activated HCAECs

**Figure 1:**
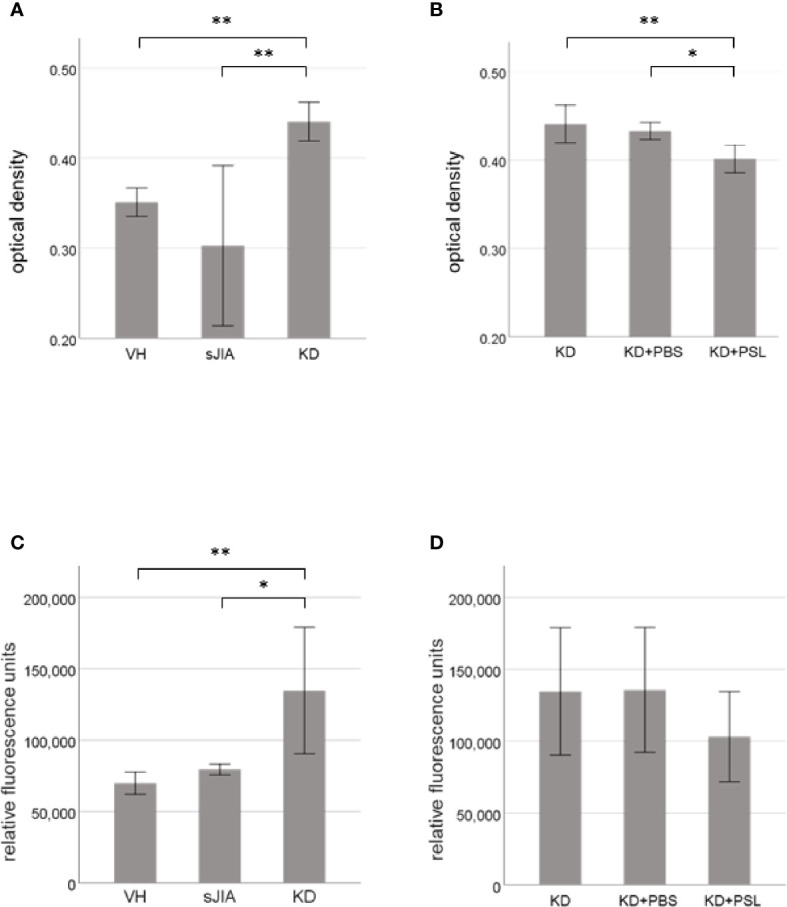
Cell proliferation and cytotoxicity of serum-activated human coronary artery endothelial cells (HCAECs). MTT and cytotoxicity assay results for HCAECs stimulated with sera from healthy controls (VH, *n* = 4) and patients with systemic juvenile idiopathic arthritis (sJIA, *n* = 4) or Kawasaki disease (KD; *n* = 8) for 24 h **(A, C)**, and serum-activated HCAECs from patients with KD (*n* = 8), and treated with PBS or prednisolone (PSL) for 24 h **(B, D)**. *P < 0.05, **P < 0.01 [one-way ANOVA followed by Bonferroni post-test **(A–C)** and Games–Howell post-test **(D)**].

MTT assay revealed that cell proliferation of serum-activated HCAECs from KD controls was significantly higher than that from the VH and sJIA groups (each *P* < 0.001) (**Figure 1A**). In KD, the proliferation of serum-activated HCAECs from the KD+PSL group was significantly lower than those of serum-activated KD controls and KD+PBS group (*P* < 0.002, and *P* = 0.012, respectively) (**Figure 1B**). Cytotoxicity in serum-activated HCAECs from KD controls was significantly higher than those in the VH (*P* = 0.007) and sJIA groups (*P* = 0.044) (**Figure 1C**). In KD, cytotoxicity of serum-activated HCAECs from the KD+PSL group tended to be lower than those of serum-activated KD controls and KD+PBS group but did not achieve statistical significance (**Figure 1D**).

### KD Serum-Activated HCAEC Supernatant HMGB-1 and sRAGE Levels and HMGB-1 Receptor Expression

**Figure 2:**
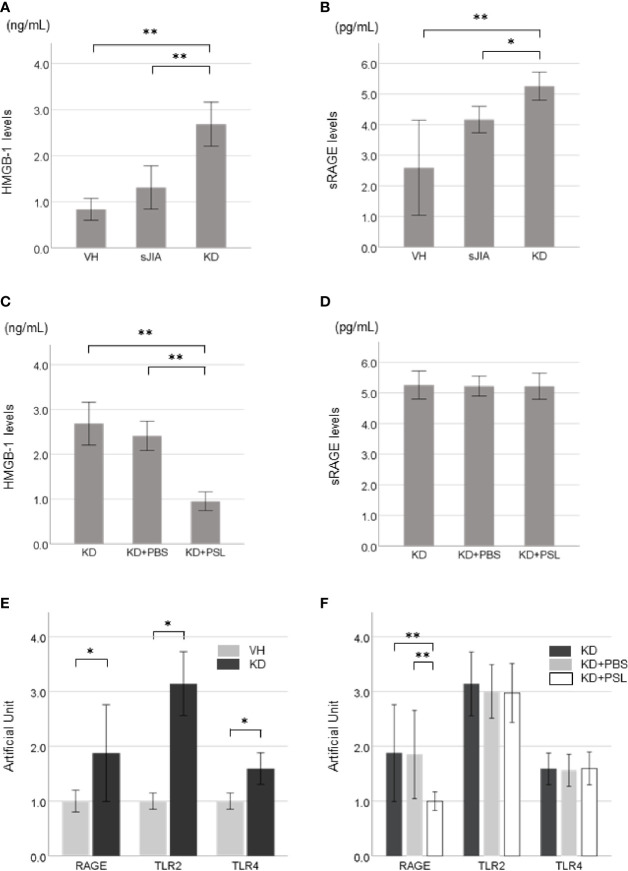
HMGB-1 and sRAGE production in Kawasaki disease (KD)-serum-activated human coronary artery endothelial cells (HCAECs). HMGB-1 levels and sRAGE levels in supernatants from **(A, B)** healthy control (VH, *n* = 4), systemic juvenile idiopathic arthritis (sJIA, *n* = 4), and KD (*n* = 8) serum-activated HCAECs and **(C, D)** KD control, KD+PBS, and KD+prednisolone (PSL) HCAECs. Expression of HMGB-1 receptors in KD serum-activated HCAECs. Artificial Unit of RAGE, TLR2, and TLR4 receptors in **(E)** VH and KD serum-activated HCAECs, and **(F)** KD control, KD+PBS, and KD+PSL HCAECs. *P < 0.05, **P < 0.01 [one-way ANOVA followed by Bonferroni post-test **(A, B, D, F)** and Games–Howell post-test **(C)**, Mann–Whitney *U*-tests **(E)**].

Supernatant HMGB-1 levels (**Figure 2A**) and sRAGE levels (**Figure 2B**) in the KD controls were significantly higher than those in the VH (each *P* < 0.001) and sJIA (*P* < 0.001 and *P* = 0.046, respectively) groups. HMGB-1 levels in the KD+PSL group were significantly lower than those in KD control and KD+PBS (each *P* < 0.001) (**Figure 2C**) groups, whereas sRAGE levels did not significantly differ between the groups (**Figure 2D**).

Basal expression levels of *RAGE*, *TLR2*, and *TLR4* receptors were increased in KD serum-activated HCAECs compared to those in VH serum-activated HCAECs (**Figure 2E**). Of KD serum-activated HCAECs, *RAGE* expression in the KD+PSL group was significantly lower than that in the KD control and KD+PBS groups (each *P* = 0.007); however, *TLR2* and *TLR4* expression did not differ significantly between the groups (**Figure 2F**).

### Immunofluorescence Staining for HMGB-1 in Serum-Activated HCAECs

**Figure 3:**
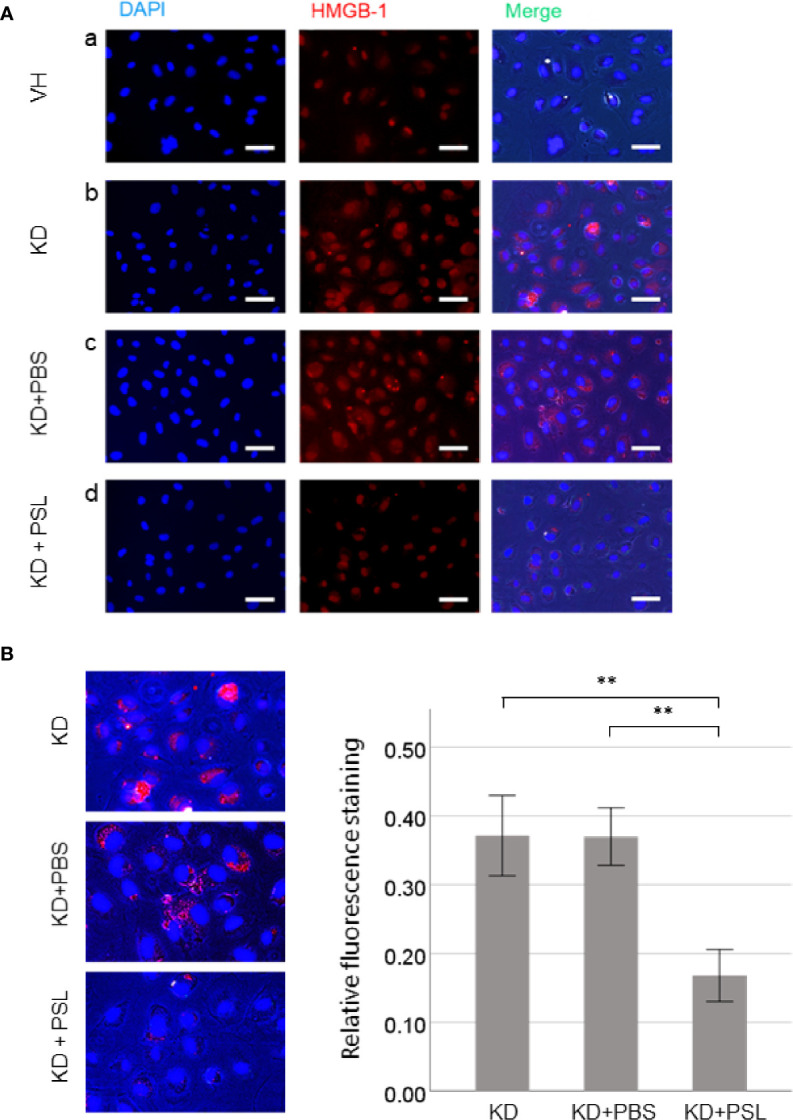
Immunostaining of HMGB-1 in serum-activated human coronary artery endothelial cells (HCAECs) (magnification, ×400). **(A)** Representative images of from healthy control (VH), Kawasaki disease (KD), and KD serum-activated HCAECs treated with PBS and prednisolone (PSL) showing DNA (blue) labelled using DAPI, HMGB-1 (red) labelled using immunofluorescence staining, and merged images. Scale bar = 100 μm. **(B)** Relative fluorescence staining of extranuclear HMGB-1 from 100 cells, selected from KD serum-activated HCAECs alone and treated with PBS and PSL. Scale bar = 100 μm. **P < 0.01 (one-way ANOVA followed by Bonferroni post-test).

Representative images revealed that serum activation of HCAECs induced HMGB-1 release from the nucleus. Compared with the VH group, KD control and KD+PBS HCAECs showed increased HMGB-1 staining in the cytoplasm or the extracellular space (**Figures 3A, a–c**). Conversely, compared with KD control and KD+PBS HCAECs, KD+PSL HCAECs showed significant reduction in cytoplasmic and extracellular HMGB-1 (**Figures 3A, d**). Quantitative analysis of the fluorescence intensity of HMGB-1 released from nucleus of serum-activated HCAECs revealed significantly lower values for KD+PSL than KD control and KD+PBS HCAECs (each *P* < 0.001) (**Figure 3B**).

### Phosphorylation of Mitogen-Activated Protein Kinase and NF-κB, and NLRP3 Inflammasome in Endothelial Cell Lysates From KD Serum-Activated HCAECs

**Figure 4:**
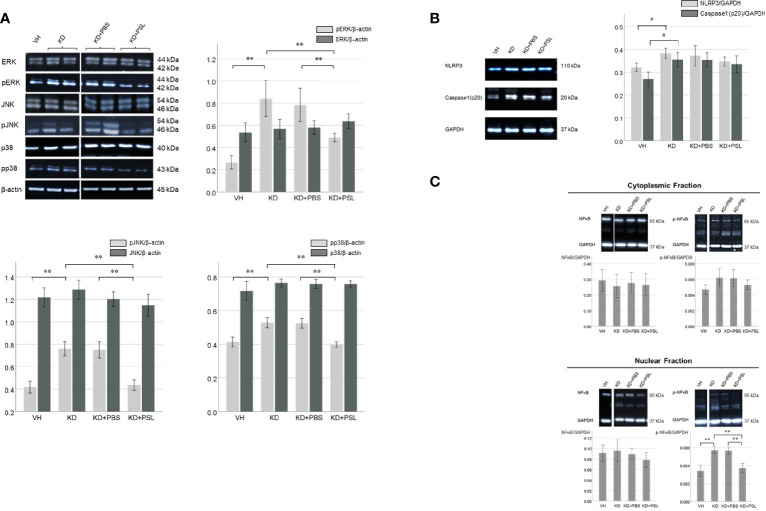
ERK1/2, JNK, p38 and NF-κB levels in serum-activated human coronary artery endothelial cells (HCAECs). **(A)** Western blot analysis of ERK1/2, pERK1/2, JNK, pJNK, p38 and pp38 from healthy controls (VH, *n* = 4), Kawasaki disease (KD, *n* = 8), KD serum-activated HCAECs treated with PBS and prednisolone (PSL) (KD+PBS and KD+PSL, *n* = 8, respectively). Bar graph shows immunoblotting results for the ERK1/2:β-actin, pERK 1/2: β-actin, JNK:β-actin, pJNK: β-actin, p38:β-actin and pp38: β-actin ratio **P < 0.01 (one-way ANOVA followed by the Bonferroni, post-test). **(B)** NLRP3 and Caspase-1(p20) in serum-activated HCAECs from healthy controls (VH, *n* = 4), KD (*n* = 8), and KD serum-activated HCAECs treated with PBS and PSL (KD+PBS and KD+PSL, *n* = 8, respectively). Bar graphs show immunoblotting results for NLRP3:GAPDH and Caspase-1(p20):GAPDH ratio in HCAEC lysates. *P < 0.05 (Kruskal–Wallis test followed by Dunn’s post-test). **(C)** Cytoplasmic and nuclear NF-κB and p-NF-κB in serum-activated HCAECs from healthy controls (VH, *n* = 4), KD (*n* = 8), and KD serum-activated HCAECs treated with PBS and PSL (KD+PBS and KD+PSL, *n* = 8, respectively). Bar graphs show immunoblotting results for NF-κB:GAPDH and p-NF-κB:GAPDH ratio in HCAEC lysates. **P < 0.01 (Kruskal–Wallis test followed by Dunn’s post-test).

To determine the role of mitogen-activated protein kinase signaling, we evaluated the levels of ERK, pERK, JNK, pJNK, p38, and pp38 in serum-activated HCAEC lysates. The ERK:β-actin, JNK:β-actin, and p38:β-actin ratios did not significantly differ between VH and KD groups; however, pERK:β-actin, pJNK:β-actin and pp38:β-actin ratios in lysates from KD controls were significantly higher than those in VH lysates (*P* < 0.001). The ERK:β-actin, JNK:β-actin, and p38:β-actin ratios did not significantly differ between KD controls, KD+PBS, and KD+PSL groups, however, pERK:β-actin, pJNK:β-actin, and pp38:β-actin ratios were significantly lower in lysates from KD+PSL groups than in lysates from the KD controls (*P* = 0.004) and KD+PBS groups (*P* = 0.006) (pERK:β-actin; *P* = 0.004 and *P* = 0.006, pJNK:β-actin; *P* < 0.001 and *P* < 0.001, pp38:β-actin; *P* = 0.002 and *P* = 0.004, respectively) (**Figure 4A**).

The NLRP3:GAPDH and cleaved caspase 1:GAPDH ratios in the lysates from KD controls were higher than those in VH lysates (*P* = 0.017 and *P* = 0.018, respectively), while the NLRP3:GAPDH and cleaved caspase 1:GAPDH ratios were lower in the lysates from KD+PSL groups compared to the lysates from the KD controls and KD+PBS; no significant difference was observed between the groups (**Figure 4B**).

The total NF-κB p65:GAPDH ratio in cell lysates did not significantly differ between the nuclear and cytoplasmic fractions of the VH group, KD controls, KD+PBS group, or KD+PSL group. Conversely, the nuclear p-NF-κB p65:GAPDH ratio in lysates from KD controls was significantly higher than those in lysates from the VH group (*P* < 0.001), whereas it was significantly reduced in the KD+PSL group compared to that in KD control and KD+PBS groups (each *P* < 0.001) (**Figure 4C**).

### IL-1β and TNF-α Production in Endothelial Cell Lysates From KD Serum-Activated Hcaecs

**Figure 5:**
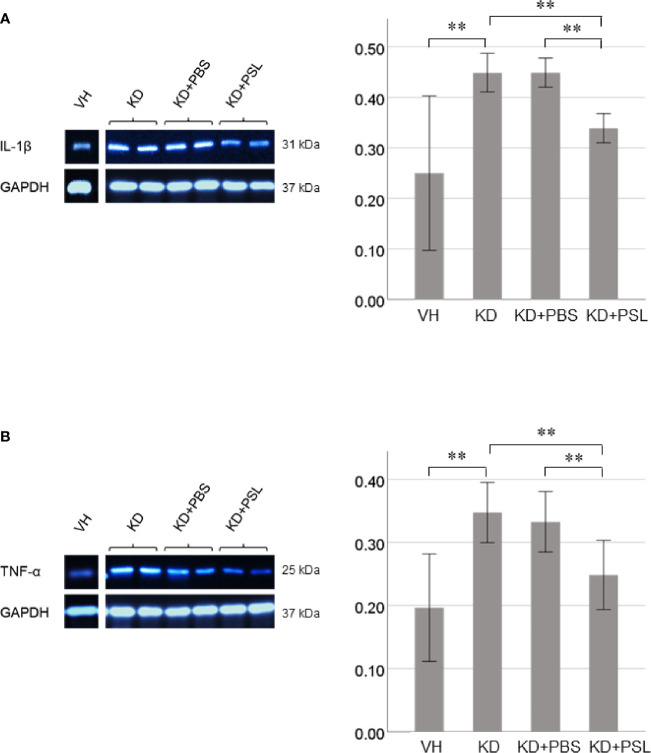
Cytokine production in serum-activated human coronary artery endothelial cell (HCAEC) lysates. Western blot analysis and quantitation of **(A)** IL-1β and **(B)** TNF-α production in serum-activated HCAEC lysates from healthy controls (VH) and Kawasaki disease (KD), and KD serum-activated HCAECs treated with PBS and prednisolone (PSL) (8 independent experiments). GAPDH was used as an internal standard. **P < 0.01 (one-way ANOVA followed by Bonferroni post-test).

Both IL-1β and TNF-α levels in lysates of KD serum-activated HCAECs were significantly higher than those from the VH group (each *P* < 0.001), whereas both were significantly lower in endothelial cell lysates from the KD+PSL group than those from KD controls and KD+PBS groups (IL-1β, each *P* < 0.001; TNF-α, *P* = 0.001, and *P* = 0.009. respectively) (**Figures 5A, B**).

## Discussion

In this study, we demonstrated that serum obtained from patients with KD prior to IVIG treatment exhibited a cytotoxic effect on HCAECs compared to that from healthy controls and patients with sJIA, in addition to triggering extracellular release of HMGB-1, up-regulating NF-κB-mediated inflammatory responses, and increasing IL-1β and TNF-α production. Although PSL treatment for KD serum-activated HCAECs did not show direct cytoprotective effects, it inhibited endothelial cell proliferation, HMGB-1 translocation, release, and downstream signaling and reduced IL-1β and TNF-α expression. These findings provide a new perspective regarding the anti-inflammatory function of PSL during the acute phase of KD.

The vasculopathic process in the acute phase of KD involves necrotizing arteries progressively destroying the arterial wall from adventia to intima, particularly the coronary arteries (22, 23). Our results are in line with this pathological process in which the KD serum induced stronger cytotoxicity to coronary endothelial cells than sJIA serum, although there was no difference in patients’ laboratory data between KD and sJIA. Since KD serum also up-regulated DAMP receptors, such as RAGE and TLRs, and induced subsequent intracellular activation of downstream ERK1/2, JNK, p38, and NF-κB signaling pathways in HCAECs, endothelial cell damage consequent to pathogenic proteins in KD serum may thus underlie several of the pathological features of KD and the effects observed during the early stages of disease progression. Specifically, during cytotoxic response to KD serum, HMGB-1 translocated from the nucleus to the extracellular space, where it may function as a DAMP or alarmin to stimulate the innate immune system and mediate inflammation in accordance with its role in the pathogenesis of delayed inflammatory responses and organ dysfunction (24).

Moreover, extracellular HMGB-1 can interact with RAGE or TLRs on the surface of inflammatory endothelial cells. HMGB-1 functional interaction with receptors activates inflammation-associated pathways and triggers a cascade of proinflammatory cytokines, including ILs, TNF-α, and macrophage inflammatory protein-1α and -1β, thereby forming a self-reinforcing inflammatory cycle (25, 26). As serum levels of HMGB-1 and S100 are elevated during the acute KD phase (7, 15–17) and RAGE activation results in up-regulated proinflammatory cytokine expression in patients with KD (18), these processes may stimulate granulocytes or endothelial cells to secrete DAMPs, thereby establishing a self-amplifying positive feedback loop. However, sRAGE, a truncated soluble form of the receptor, acts as a decoy and prevents the RAGE activation-mediated inflammatory response (27). Consistent with our results, Wittkowski et al. (28) found that sRAGE levels in acute KD were significantly lower than those post-IVIG or in the subacute phase, suggesting the potential anti-inflammatory effect of sRAGE on inflammatory vascular disorders. Additionally, subsequent activation of HMGB-1/RAGE-specific downstream signaling pathways and increased levels of IL-1β or TNF-α constitute parallel or consecutive events in response to increased HMGB-1 levels. The HMGB-1/RAGE signaling pathway in endothelial cells also induces cathepsin B activation, subsequently inducing canonical pyroptosis *via* NLRP3 inflammasomes in KD vasculitis (18). Therefore, our study also supports the hypothesis that extracellular HMGB-1 is possibly up-regulated to act as a functional cytokine influencing inflammation and the innate immune response, and that it may thus contribute to the pathogenesis of KD vasculitis (29, 30).

We further revealed that PSL treatment of KD serum-activated HCAECs inhibited extracellular HMGB-1 release, reduced *RAGE* expression, and inhibited NF-κB-mediated inflammatory responses, in addition to reducing IL-1β and TNF-α production. Conversely, PSL treatment did not show cytoprotective effects or sRAGE up-regulation in KD-serum-activated HCAECs, supporting that the effects of PSL treatment might occur by directly inhibiting extracellular HMGB-1 release. In general, the anti-inflammatory effects of glucocorticoids are attributable to the transcriptional effects of glucocorticoid-receptor agonism, which alters the transcription of numerous genes both positively and negatively by targeting specific cell populations to combat the immune system hyperactivation or systemic infections (10, 31–33). Glucocorticoid treatment inhibits the expression of proinflammatory genes, including NF-κB and activator protein 1 (10); therefore, the observed PSL-induced inhibition of HMGB-1 release might occur *via* NF-κB signaling.

Another possible mechanism is that glucocorticoids inhibit TNF-α synthesis in activated monocytes/macrophages, a process at least partially involved in the anti-inflammatory effects of TNF-α-induced HMGB-1 secretion from endothelial cells (30). Therefore, PSL treatment of KD serum-activated HCAECs likely has an upstream regulatory component and potentially inhibits the HMGB-1 signaling pathway. CD14 is important in efficient HMGB-1-dependent TLR activation (34, 35), whereas RAGE offers a different transduction pathway in providing a transport route for HMGB-1 and its partner molecule complexes by endocytosis to the endolysosomal component (36). Rather than being degraded in the lysosomes, HMGB-1 transported molecules then leak out from the permeabilized lysosomes into the cytosol to reach and activate cognate cytoplasmic receptors, thereby causing inflammation (36). In the present study, we could not confirm a functional role for the HMGB-1/TLRs signaling pathway and HMGB-1 induced NLRP3 inflammasome activation through the serum stimulation experiments using cultured HCAECs owing to the lack of lymphocytes and macrophages. However, our findings suggest that PSL application during acute KD has a distinct potential to also ameliorate HMGB-1/RAGE-mediated inflammatory responses in KD vasculitis, which is borne out by the efficacy of glucocorticoids at reducing the incidence of CAA and the number of IVIG non-responders in KD (4–6).

Nevertheless, there were several limitations in this study with respect to the effects of PSL treatment in KD vasculitis that remain unaddressed. Serum samples from patients with KD used in this study were limited; hence, we did not measure cytokine and inflammatory markers other than those from routine blood testing. Glucocorticoids affect virtually all immune cells and their precise effects depend on the differentiation and activation state of the cell, making interpretation of *in vivo* effects in specific populations difficult. Glucocorticoids inhibit neutrophil-dependent endothelial cell injury (37) or platelet–neutrophil aggregate formation (38), thereby reducing cytokine-induced adhesion and inhibiting amplified reciprocal vascular inflammatory activation. However, we could not examine the contribution of neutrophil-, monocyte-, and platelet-dependent endothelial cell activation since our serum-stimulation experiments were performed using cultured coronary endothelial cells. Given the differences in glucocorticoid-receptor levels between endothelial cells and various vascular beds, the relative proportion of specific glucocorticoid-receptor isoforms in tissues and cells may influence their responses to glucocorticoid treatment (39–41). IVIG is the standard and most effective treatment for KD, however, the present study focused on the mechanism of action of corticosteroids in KD; and hence no examination or analysis of the effects of IVIG was conducted in this study.

In conclusion, our findings suggest that extracellular HMGB-1 is potentially up-regulated to act as a functional cytokine with roles in both inflammation and the innate immune response, thereby mediating KD pathogenesis. Treatment with PSL during the acute phase of KD ameliorates HMGB-1/RAGE-mediated inflammatory responses and reduces IL-1β and TNF-α production. Inhibiting extracellular HMGB-1 may also inhibit the over-activated innate immune system, thus offering potential relief from or prevention of severe KD vasculitis.

## Data Availability Statement

The datasets presented in this study can be found in online repositories. The names of the repositories and accession numbers can be found in the article/**Supplementary Material**.

## Ethics Statement

The studies involving human participants were reviewed and approved by Kagoshima University and Kagoshima City Hospital Ethics Committee. Written informed consent to participate in this study was provided by the participants’ legal guardian/next of kin. Written informed consent was obtained from the individual(s), and minor(s)’ legal guardian/next of kin, for the publication of any potentially identifiable images or data included in this article.

## Author Contributions

KU, YN and YK conceived and designed the experiments. KU and YM performed the experiments. KU and YN analyzed the data and designed the figures. KU performed the statistical analysis and wrote the first draft of the manuscript. All authors contributed to the article and approved the submitted version.

## Funding

This work was supported financially by a Japan Society for the Promotion of Science (JSPS) KAKENHI Grant Numbers JP16K19657 and JP19K08302.

## Conflict of Interest

The authors declare that the research was conducted in the absence of any commercial or financial relationships that could be construed as a potential conflict of interest.

## Abbreviations

KD, Kawasaki disease; HMGB-1, high mobility group box-1; PSL, Prednisolone; sJIA, systemic juvenile idiopathic arthritis; HCAECs, human coronary artery endothelial cells; ERK, extracellular signal-regulated kinase; JNK, c-Jun N-terminal kinase; NF-κB, nuclear factor kappa B; NLRP3, Nucleotide-binding oligomerization domain-like receptor family, pyrin domain-containing 3; IL, interleukin; TNF, tumor necrosis factor; CAA, coronary artery abnormalities; IVIG, intravenous immunoglobulin; DAMPs, damage-associated molecular pattern; RAGE, receptor for advanced glycation end products; TLRs, Toll-like receptors; PBS, phosphate buffered saline; MTT, 3-(4,5-dimethylthiazol-2-yl)-2,5-diphenyl tetrazolium bromide; GAPDH, glyceraldehyde-3-phosphate dehydrogenase; RT-PCR, reverse transcription–polymerase chain reaction.
