# Supplementary figures and images for: Prednisolone Suppresses the Extracellular Release of HMGB-1 and Associated Inflammatory Pathways in Kawasaki Disease
Source: Front Immunol. 2021 May 17;12:640315. doi: 10.3389/fimmu.2021.640315 (PMC8165186; doi:10.3389/fimmu.2021.640315)

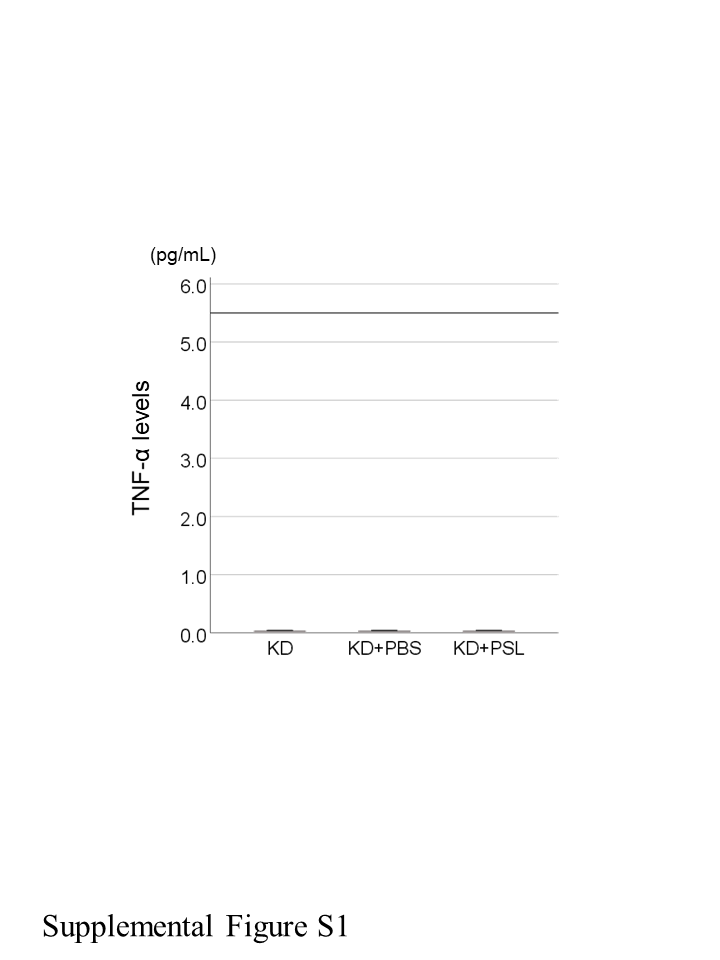

Supplement: Supplementary file 1 [file Image_1.tif]

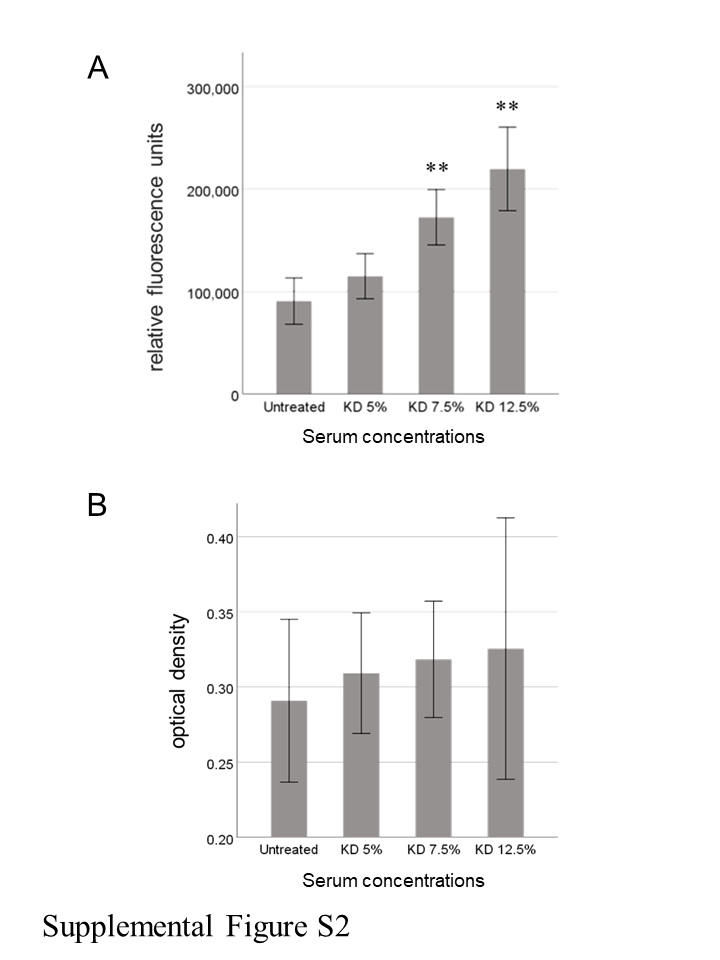

Supplement: Supplementary file 2 [file Image_2.tif]
